# Equine bone marrow MSC‐derived extracellular vesicles mitigate the inflammatory effects of interleukin‐1β on navicular tissues in vitro

**DOI:** 10.1111/evj.14090

**Published:** 2024-04-08

**Authors:** Vivian G. Quam, Zarah A. Belacic, Sidney Long, Hilary C. Rice, Madhu S. Dhar, Sushmitha Durgam

**Affiliations:** ^1^ Department of Veterinary Clinical Sciences, College of Veterinary Medicine The Ohio State University Columbus Ohio USA; ^2^ Ballarat Veterinary Practice Equine Clinic Miners Rest Victoria Australia; ^3^ Department of Large Animal Clinical Sciences, College of Veterinary Medicine University of Tennessee Knoxville Tennessee USA

**Keywords:** bone marrow MSC, deep digital flexor tendon, extracellular vesicles, horse, navicular fibrocartilage

## Abstract

**Background:**

Safe, efficacious therapy for treating degenerate deep digital flexor tendon (DDFT) and navicular bone fibrocartilage (NBF) in navicular horses is critically necessary. While archetypal orthobiologic therapies for navicular disease are used empirically, their safety and efficacy are unknown. Mesenchymal stem cell‐derived extracellular vesicles (EV) may overcome several limitations of current orthobiologic therapies.

**Objectives:**

To (1) characterise cytokine and growth factor profiles of equine bone marrow mesenchymal stem cell (BM‐MSC)‐derived extracellular vesicles (BM‐EV) and (2) evaluate the in vitro anti‐inflammatory and extracellular matrix (ECM) protective potentials of BM‐EV on DDFT and NBF explant co‐cultures in an IL‐1β inflammatory environment.

**Study design:**

In vitro experimental study.

**Methods:**

Cytokines (IL‐1β, IL‐6, IL‐10, IL‐1ra and TNF‐α) and growth factors (TGFβ1, VEGF, IGF1 and PDGF) in equine BM‐EV isolated via ultracentrifugation and precipitation methods were profiled. Forelimb DDFT and NBF explant co‐cultures from seven horses were exposed to media alone, or media containing 2 × 10^9^ ± 0.1 × 10^9^ particles/mL or 10 μg/mL BM‐EV (BM‐EV), 10 ng/mL interleukin‐1β (IL‐1β), or IL‐1β + BM‐EV for 48 h. Co‐culture media IL‐6, TNF‐α, MMP‐3, MMP‐13 concentrations and explant sulphated glycosaminoglycan (sGAG) content were quantified.

**Results:**

IL‐6, IGF1 and VEGF concentrations were 102.1 (37.61–256.2) and 182.3 (163.1–226.3), 72.3 (8–175.6) and 2.4 (0.1–2.6), 108.3 (38.3–709.1) and 211.4 (189.1–318.2) pg/mL per 2 × 10^9^ ± 0.1 × 10^9^ particles/mL or 10 μg/mL 10 μg of BM‐EV isolated via ultracentrifugation and precipitation methods, respectively. Co‐culture media MMP‐3 in BM‐EV‐ (*p* = 0.03) and BM‐EV + IL‐1β‐treated (*p* = 0.01) groups were significantly lower than the respective media and IL‐1β groups. DDFT explant sGAG content of BM‐EV (*p* = 0.003) and BM‐EV + IL‐1β groups were significantly higher compared with IL‐1β group.

**Main limitations:**

Specimen numbers are limited, in vitro model may not replicate clinical case conditions, lack of non‐MSC‐derived EV control group.

**Conclusions:**

Equine BM‐EV contains IL‐6 and growth factors, IGF1 and VEGF. The anti‐inflammatory and ECM protective potentials of BM‐EV were evident as increased IL‐6 and decreased MMP‐3 concentrations in the DDFT‐NBF explant co‐culture media. These results support further evaluation of BM‐EV as an acellular and ‘off‐the‐shelf’ intra‐bursal/intrasynovial therapy for navicular pathologies.

## 1 INTRODUCTION

Widespread use of magnetic resonance imaging (MRI) has demonstrated that deep digital flexor tendon (DDFT) and palmar navicular bone fibrocartilage (NBF) pathologies are largely responsible for the clinical signs associated with navicular disease in horses.^1^, ^2^, ^3^ Unfortunately, these tissues have poor innate healing capacity and degenerative changes often ensue following injury.^1^, ^4^, ^5^, ^6^, ^7^ To date, intra‐synovial/bursal corticosteroid injections are a mainstay in the clinical management of navicular disease due to their potent anti‐inflammatory properties, despite their controversial effects on tissue catabolism.^2^, ^8^ The optimal local therapy would provide beneficial symptom‐modifying effects as well as improve structural healing characteristics for the multitude of structures (DDFT and NBF, navicular suspensory apparatus, navicular bursitis and/or distal interphalangeal joint) affected in navicular disease. Orthobiologics, including mesenchymal stem cell therapies, are gaining traction for their demonstrated anti‐inflammatory, immunomodulatory and trophic effects on endogenous musculoskeletal tissue cells.^9^, ^10^, ^11^

Autologous and allogenic bone marrow mesenchymal stem cells (BM‐MSC) have been assessed in experimental and naturally occurring osteoarthritis and tendinopathy.^12^, ^13^, ^14^, ^15^, ^16^ Documented benefits of local BM‐MSC treatments include reducing tendon reinjury rates,^14^ improving tendon and cartilage histological structure, increasing biomechanical properties and reducing tissue inflammation during healing,^13^, ^16^, ^17^, ^18^ and an overall improvement in lameness.^12^, ^14^, ^15^ Further investigations are needed to understand the mechanisms of action leading to these favourable outcomes. Additionally, there are barriers to BM‐MSC use in clinical practice. Donor site morbidity and the lagtime between bone marrow harvest and treatment needed for laboratory processing have to be considered with autologous BM‐MSC, while immunogenicity concerns exist with allogenic BM‐MSC.^12^, ^15^, ^19^, ^20^, ^21^ Furthermore, BM‐MSC culture supplements such as fetal bovine serum (FBS), which is necessary to preserve cell viability during transportation of BM‐MSC, have produced adverse effects in horses. Potential negative interactions with co‐administered medications such as sedatives, local anaesthetics, antibiotics and corticosteroids may also preclude ‘point of care’ intralesional/local BM‐MSC treatment.^19^, ^21^

Extracellular vesicles (EV) are membrane‐bound vesicles secreted via exocytosis. EV, which include exosomes (30–120 nm) and microvesicles (80–1000 nm), are storehouses of cytokines, growth factors, mRNA and microRNA reflective of the parent cells.^22^, ^23^ The EV secreted by BM‐MSC, henceforth referred to as BM‐EV, have demonstrated anti‐inflammatory and immunomodulatory benefits, as well as participation in cell‐to‐cell interactions and protein/peptide delivery to recipient cells both in vitro and in vivo.^24^, ^25^, ^26^, ^27^, ^28^ Recent in vitro studies have reported that equine BM‐EV decreased gene expression of extracellular matrix (ECM) degradative enzymes and increased chondrocyte proliferation.^29^, ^30^ In rodent and rabbit in vivo experimental tendon/ligament and articular cartilage injury models, BM‐EV reduced inflammation and improved the histological structure of the tissues, respectively.^31^, ^32^, ^33^ Thus, BM‐EV may share the known benefits of BM‐MSC therapy for tendon and cartilage healing, while overcoming several aforementioned practical limitations of BM‐MSC. As EV are free of cellular material, immunogenicity concerns are eliminated, in addition to mitigating concerns of donor site morbidity and culture lagtime. Before affirming clinical relevance, further investigation of equine‐specific BM‐EV is necessary including characterisation of the proteome, growth factor and cytokine contents.

In the context of equine navicular disease, BM‐EV conceivably comprises an ‘off‐the‐shelf’ cell‐free therapy for intra‐synovial/intra‐bursal treatment. The objectives of this study were two‐fold. First, the cytokine and tissue anabolic growth factor concentrations in equine BM‐EV were quantified. Secondly, the anti‐inflammatory and ECM protective effects of equine BM‐EV on forelimb DDFT‐NBF co‐cultures in an IL‐1β‐induced in vitro inflammation were assessed.

## 2 MATERIALS AND METHODS

### 2.1 Equine BM‐MSC isolation

Equine BM‐MSC was isolated using standard protocols as described previously.^12^, ^34^ Fetal bovine serum (FBS, Gibco™) from a single lot was purchased and used for BM‐MSC culture/expansion and all experiments described in this study. Bone marrow aspirates were collected from the sternum of 7 horses (Table S1) by use of a bone marrow biopsy needle (Jamshidi™, BD). Approximately 12–15 mL of bone marrow was aspirated into syringes containing 1000 U of heparin. Each bone marrow aspirate was diluted with 15 mL of PBS solution and centrifuged at 300*g* for 10 min. The supernatant was removed, the pellet was resuspended in PBS solution and centrifugation was repeated. Pelleted cells were then resuspended in 12 mL of Dulbecco's Modified Eagles Medium (DMEM; Gibco™) supplemented with 10% FBS, 300 μg of l‐glutamine/mL (Gibco™), 100 U of sodium penicillin/mL and 100 μg of streptomycin sulphate/mL (Gibco™) (basal medium). In one of the seven horses, mononuclear cells were first isolated from bone marrow aspirates via density gradient centrifugation (Ficoll‐Paque PREMIUM 1.078).^12^ Resuspended cells were placed in 75‐cm^2^ cell culture flasks and incubated at 37°C in a 5% carbon dioxide atmosphere with 90% humidity. The resultant BM‐MSC were passaged after discernible colony‐forming units (>50 cells) were visualised. BM‐MSC were subsequently plated at 10 000 cells/cm^2^, cultured in basal medium until 70% confluency and underwent one additional passage. All seven horses from which BM‐MSCs were isolated for experiments in this study were apparently healthy.

### 2.2 Equine BM‐EV isolation

Once passage 2 BM‐MSC reached 50% confluence, basal medium was replaced with DMEM supplemented with 10% EV‐depleted FBS, 300 μg of l‐glutamine/mL, 100 U of sodium penicillin/mL and 100 μg of streptomycin sulphate/mL 48 h before BM‐EV isolation.^35^, ^36^ EV‐depleted FBS was prepared via ultracentrifugation of FBS at 100 000*g* for 18 h at 4°C using an Optima L‐80XP ultracentrifuge and SW41Ti swinging‐bucket rotor (both from Beckman–Coulter).^36^ The cell culture supernatant was collected from BM‐MSC every 48 h for BM‐EV isolation.^35^, ^37^ The BM‐MSC cultures were replenished with fresh DMEM supplemented with 10% EV‐depleted FBS, 300 μg of l‐glutamine/mL, 100 U of sodium penicillin/mL and 100 μg of streptomycin sulphate/mL and the process was continued until the BM‐MSC reached 95% confluency. BM‐EV was isolated from the second to fifth passage BM‐MSC in a similar manner.

#### 2.2.1 Ultracentrifugation method

The cell culture supernatant was first centrifuged at 2000*g* for 30 min at 4°C to remove cellular debris. Next, the cell culture supernatant underwent ultracentrifugation at 100 000*g* for 2 h at 4°C in an Optima L‐80XP ultracentrifuge and SW41Ti swinging‐bucket rotor (both from Beckman–Coulter).^35^, ^38^ The resultant pellet from every 10 mL of cell culture supernatant was resuspended in 100 μL of PBS containing protease inhibitor (EDTA‐free cOmplete™, Roche) and stored at −80°C until subsequent assays. Flow cytometry confirmed CD9 surface marker expression in BM‐EV isolates, with a mean fluorescence intensity (MFI) ± SD value of 1.2 ± 0.32.^32^

#### 2.2.2 Precipitation method

The cell culture supernatant was first centrifuged at 2000*g* for 30 min at 4°C to remove cellular debris. Then, 0.5 volume of Total Exosome Isolation reagent (Life Technologies Inc.™) was added to the cell culture supernatant collected in 50 mL conical centrifuge tubes (Corning™ Falcon™), gently mixed and incubated at 2°C–8°C overnight.^37^ Next, the cell culture supernatant mixture was further centrifuged at 10 000*g* for 1 h at 4°C in an Optima L‐80XP ultracentrifuge and SW41Ti swinging‐bucket rotor (both from Beckman–Coulter). The resultant pellet from every 10 mL of cell culture supernatant mixture was resuspended in 100 μL of PBS containing protease inhibitor and stored at −80°C until subsequent assays. Flow cytometry confirmed CD9 surface marker expression in BM‐EV isolates, with a MFI ± SD value of 1.45 ± 0.21.^32^

### 2.3 BM‐EV transmission electron microscopy (TEM) assessment

A 100 μL aliquot of representative BM‐EV isolated via ultracentrifugation and precipitation methods was separately fixed with 500 μL of 2% paraformaldehyde (Electron Microscopy Sciences) for 5 min. Ten microlitres of BM‐EV solution was loaded on formvar/carbon film coated 200 mesh copper EM grids and incubated for 1 min. The grid was then stained with 250 μL of filtered 1% uranyl acetate solution on the surface of the EM grid. The excess stain was removed by blotting the edge of the grid with filter paper. Tecnai G2‐30 transmission electron microscope was used to observe the grids at ×250 000 magnification.

### 2.4 BM‐EV protein quantification

Protein concentration was measured via Pierce™ BCA Protein Assay Kit (ThermoFisher Scientific) following the manufacturer's protocol. A 25 μL aliquot of BM‐EV was combined with 25 μL RIPA lysis buffer (ThermoFisher Scientific) containing protease inhibitor. A 9 μL aliquot of the resultant sample was pipetted into a microplate in duplicate, and 260 μL of the working reagent was added to each well. The plate was then read at 562 nm, and absorbance values were compared with the reference standard curve prepared with bovine serum albumin.

### 2.5 Nanoparticle tracking analysis

BM‐EV isolated via ultracentrifugation and precipitation methods were diluted 1:50 in PBS. BM‐EV concentration and size distribution were measured using Malvern NanoSight NS300 (ATA Scientific Instruments) five times during a 45 s video, and data were obtained via NTA 3.2 software. Three replicates from pooled BM‐EV isolated via ultracentrifugation and precipitation were analysed.

### 2.6 BM‐EV cytokine and growth factor ELISA analyses

Cytokine (IL‐1β, IL‐6, IL‐10, IL‐1ra and TNF‐α) and growth factor (TGFβ1, VEGF, IGF‐1 and PDGF) concentrations were individually determined via equine‐specific ELISA kits in accordance with the manufacturer's protocol. Briefly, 10 μg concentration of pooled BM‐EV aliquots from passage 2–5 BM‐MSC (as determined by the BCA Protein Assay) from individual horses were used for ELISA. BM‐EV isolated from ultracentrifugation and precipitation methods from individual horses were used and analysed in duplicates. The 10 μg concentration of BM‐EV was combined with an equal volume of RIPA lysis buffer, the volume adjusted to 100 μL and then combined with the corresponding cytokine or growth factor conjugate. The samples were then washed and incubated with the substrate solution, and the optical density was measured with a microplate reader set at 450 nm and compared with the reference standard curve.

### 2.7 Forelimb DDFT and NBF explant harvest

DDFT and NBF explants were aseptically collected from the forelimbs of seven horses (Table S2; aged 4–12 years and donated to the university's post‐mortem research efforts) immediately following euthanasia with sodium pentobarbital (150 mg/kg i.v.).^39^ Based on preliminary experiments and previous in vitro studies with navicular tissues and cells conducted by our group,^39^, ^40^, ^41^ and an expected minimum 20% difference between experimental groups IL‐1b and BM‐EV + IL‐1b with a power calculation at >0.8, the sample size was estimated at *n* = 8. Using aseptic technique, the navicular bone was dissected en‐bloc from the foot. The DDFT one‐half centimetre proximal to and directly opposing the navicular bone was also harvested using aseptic technique. The DDFT and opposing NBF were determined to be normal based on gross assessment at the time of tissue harvest. Two‐millimetre‐thick tissue explants were prepared from DDFT and NBF and harvested using a 4‐mm dermal biopsy punch, as described previously.^39^ On average, about 10–12 DDFT and NBF explants were collected from each forelimb. Explants were allowed to equilibrate to culture conditions (37°C with 5% CO_2_ at 95% humidity) for 48 h in DMEM containing 4.5 g/L glucose and l‐glutamine (300 μg/mL) supplemented with sodium penicillin (100 U/mL), streptomycin sulphate (100 μg/mL) and l‐ascorbic acid (50 μg/mL) or ‘basal medium’ before the initiation of experimental treatments.

### 2.8 DDFT and NBF co‐culture setup and experimental groups

Following equilibration, individual DDFT and NBF explants were transferred to 24‐well polystyrene transwell plates (Corning) with inserts lined with polyester porous membranes (diameter, 12 mm; pore size, 3 μm). The DDFT explant was placed at the bottom with the flexor surface facing upwards, and NBF explant was suspended in the well insert such that the flexor surface faced the DDFT explant. Each transwell with DDFT and NBF explants was filled with 1 mL of basal medium and allowed to equilibrate for an additional 24 h.

Following acclimatisation, DDFT‐NBF explant co‐cultures were treated with an equivalent volume of fresh basal medium (referred to as ‘media’), media containing 2 × 10^9^ ± 0.1 × 10^9^ particles/mL BM‐EV (referred to as ‘BM‐EV’), media containing 10 ng/mL of recombinant equine IL‐1β (referred to as ‘IL‐1β’), or media containing 2 × 10^9^ ± 0.1 × 10^9^ particles/mL BM‐EV and 10 ng/mL of IL‐1β (referred to as ‘BM‐EV + IL‐1β’). This concentration of BM‐EV was chosen based on published literature focused on extracellular vesicles for musculoskeletal regeneration^35^, ^38^ and represented 10 μg/mL protein concentration as determined by the BCA assay. All experimental groups were evaluated in duplicate DDFT‐NBF co‐cultures in each horse. The BM‐EV used in all co‐culture experiments were isolated via precipitation method and pooled from passage 2–5 BM‐MSC of 1 apparently healthy horse (CD90+, vimentin positive, CD34^−^, CD45^−^ and MHC‐II negative) evaluated for allogeneic intralesional and intraarticular injections in a previously published retrospective study.^12^ The co‐cultures with the respective treatments were maintained for 48 h before sample collection for downstream assays.

The co‐culture media from individual wells was collected, pooled and aliquoted in 250 μL volumes, and stored at −80°C for follow‐up analyses. The DDFT and NBF explants were placed in 1.5 mL microcentrifuge tubes, rinsed with PBS and individually snap frozen in liquid nitrogen, and stored at −80°C until sulphated glycosaminoglycan (sGAG) quantification.

### 2.9 Assessment of anti‐inflammatory and ECM protective properties of BM‐EV on DDFT‐NBF co‐cultures

#### 2.9.1 Co‐culture media TNFα and IL‐6 concentrations

The anti‐inflammatory effect of BM‐EV was assessed by measuring TNF‐α and IL‐6 levels in the co‐culture media samples using equine‐specific R&D Systems DuoSet ELISA in accordance with the manufacturer's protocol.^42^

#### 2.9.2 Co‐culture media MMP‐3 and MMP‐13 concentrations

ECM protective property of BM‐EV was evaluated by measuring MMP‐3 and ‐13 levels in the co‐culture media samples using equine‐specific ELISAs in accordance with the manufacturer's protocol. For all ELISA, 100 μL volume of co‐culture media from each experimental group of individual horses was assessed in duplicate.

#### 2.9.3 Explant sGAG quantification

The sGAG content of DDFT and NBF explants was quantified via dimethylmethylene blue binding (DMMB) assay.^43^ For the DMMB assay, the cryopreserved DDFT and NBF explants (2 explants/experimental group/horse) were thawed and individually weighed. Every 10 mg of DDFT or NBF explant was digested in 1 mL of 0.5 mg/mL concentration papain (ThermoFisher Scientific) overnight at 65°C.^44^ All values were compared against a standard curve prepared using shark chondroitin sulphate values to estimate sGAG content in paired replicates of explants. Undiluted standard curve and 50 μL of explant digests were combined with 200 μL of 1,9‐DMMB dye and ethyl alcohol substrate. After gentle mixing, the light absorbance of each well was determined at 530 nm.

The total DNA content in papain digests from DDFT and NBF explants (used for sGAG measurement) was determined via fluorometric measurement of Hoechst 33258 (Sigma) dye incorporation.^45^ The sGAG contents of DDFT and NBF explants were normalised with the DNA contents measured in the respective papain digests.

#### 2.9.4 Data analysis

Data normality was assessed using the Shapiro–Wilk test. Total protein concentrations (as determined by BCA assay), cytokine and growth factor profiles of BM‐EV isolated via ultracentrifugation and precipitation methods are compared via Mann–Whitney *U*‐test. All outcome measures from co‐culture experiments under treatment conditions media, BM‐EV, IL‐1β or BM‐EV + IL‐1β were compared using ANOVA or the analogous Kruskal–Wallis test for non‐parametric data. Post hoc analyses with pair‐wise comparisons were conducted with Tukey or Dunn test for parametric and non‐parametric data, respectively. Data are expressed as median with interquartile ranges or mean and SD, and the difference between groups is considered significant at *p* ≤ 0.05. Statistical analysis was performed using R Core Team 2021 and the figures were produced using GraphPad Prism Version 9.

## 3 RESULTS

### 3.1 BM‐EV size and concentration

**FIGURE 1:**
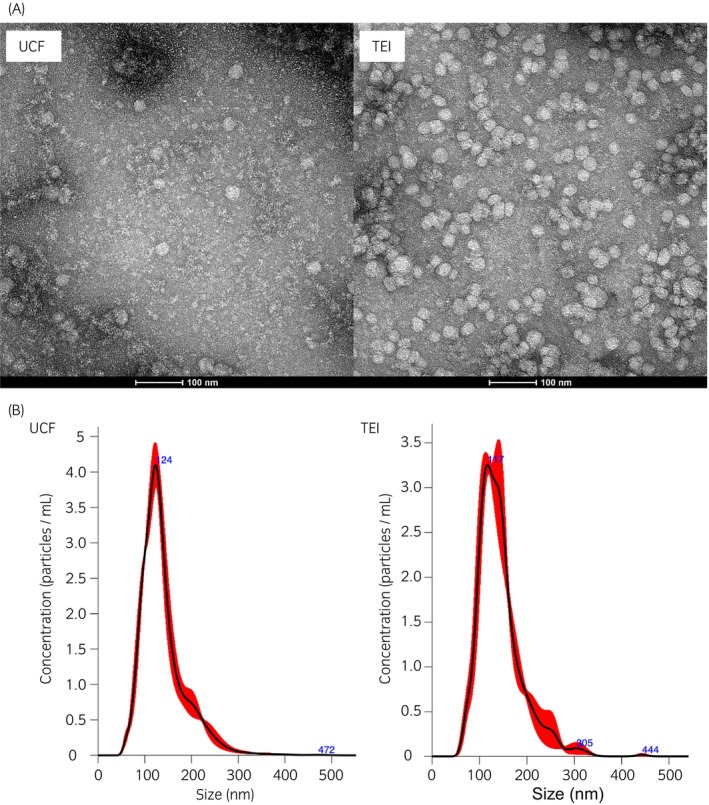
(A) Representative negative‐stained transmission electron microphotographs of BM‐EV isolated via ultracentrifugation (UCF) and precipitation (Total Exosome Isolation reagent, TEI) methods. (B) Representative histogram data from nanoparticle tracking analyses of BM‐EV isolated via ultracentrifugation (UCF) and precipitation (Total Exosome Isolation reagent, TEI) methods.

**FIGURE 2:**
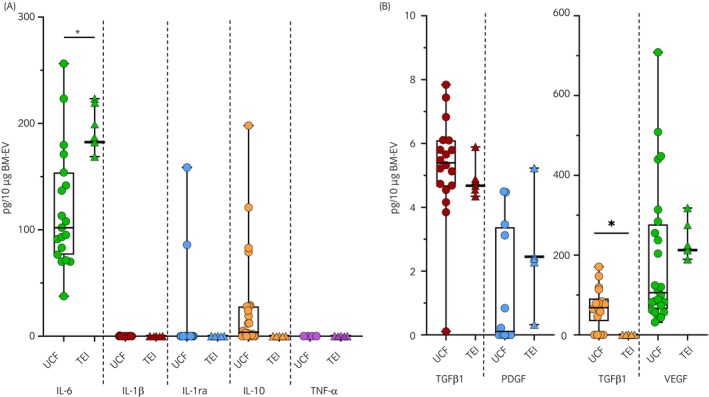
(A) Cytokines IL‐6, IL‐1β, IL‐1ra, IL‐10, TNFα and (B) Growth factors TGFβ1, PDGF, IGF1, VEGF (median and interquartile range, IQR) in 2 × 10^9^ ± 0.1 × 10^9^ particles/mL or 10 μg of BM‐EV isolated via ultracentrifugation (UCF) or precipitation (TEI) methods. Significant differences in cytokines and growth factors between isolation methods depicted via bars and asterisks.

TEM confirmed that BM‐EV were isolated from both ultracentrifugation and precipitation methods (Figure 1A). The median and interquartile ranges (IQR) of BM‐EV size as measured on the TEM images from ultracentrifugation (80.5, 42–120 nm) and precipitation (92, 50–150 nm) methods were not significantly different (*p* = 0.3). Nanosight analyses demonstrated that the size of BM‐EV (mean ± SD) isolated from ultracentrifugation and precipitation were 138.9 + 51.2 nm and 143.3 + 47.9 nm, respectively (*p* = 0.1). The concentration of BM‐EV isolated via ultracentrifugation (1.57 × 10^10^ ± 0.11 × 10^10^ particles/mL) did not significantly differ (*p* = 0.1) from that isolated via precipitation (1.43 × 10^10^ ± 0.47 × 10^10^ particles/mL). Representative histograms generated from Nanosight analyses are depicted in Figure 2B. The mean and SD of BM‐EV protein concentration as measured via BCA assay from the ultracentrifugation method (849.3 ± 72 μg/mL) was significantly (*p* = 0.01) lower than from the precipitation method (1218.6 ± 56 μg/mL).

### 3.2 BM‐EV cytokine profile

Median and IQR of IL‐6, IL‐1β, IL‐1ra, IL‐10 and TNFα concentrations per 2 × 10^9^ ± 0.1 × 10^9^ particles/mL or 10 μg/mL of BM‐EV are depicted in Figure 2A. Among the cytokines assayed, IL‐6 was detected in the highest concentration with a median of 102.1 (37.61–256.2) pg and 182.3 (163.1–226.34) pg/mL per 10 μg of BM‐EV isolated via ultracentrifugation and precipitation methods, respectively. The assayed values of IL‐1β, IL‐1ra, IL‐10 and TNFα in both isolation methods were less than the lowest detection limit of the respective ELISA.

### 3.3 BM‐EV tissue anabolic growth factor profile

Median and IQR of TGFβ1, PDGF, IGF1 and VEGF concentrations per 2 × 10^9^ ± 0.1 × 10^9^ particles/mL or 10 μg/mL of BM‐EV are depicted in Figure 2B. A wide range in TGFβ1 and VEGF concentrations in BM‐EV isolated via ultracentrifugation and precipitation methods were noted; with no significant differences in the TGFβ1 (*p* = 0.2) and VEGF (*p* = 0.1) concentrations between the two isolation methods. In the majority of BM‐EV samples isolated with both methods, PDGF and IGF1 concentrations were less than the lowest detection limits of the respective ELISA.

### 3.4 Anti‐inflammatory effect of BM‐EV on DDFT‐NBF co‐cultures

**FIGURE 3:**
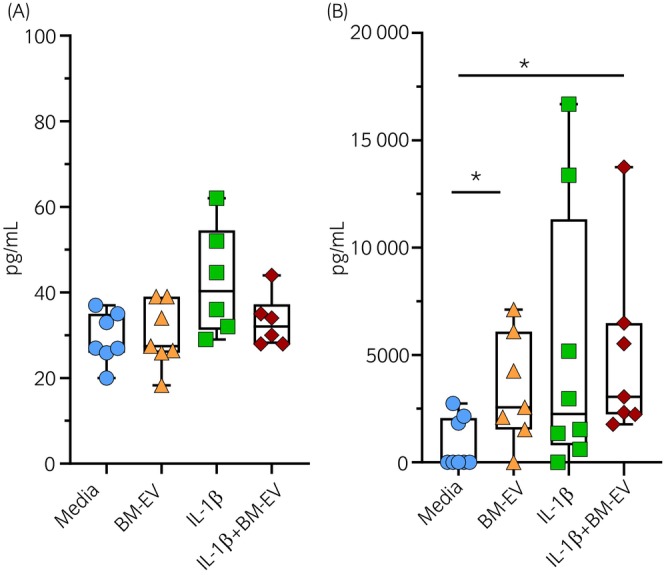
(A) TNFα and (B) IL‐6 concentrations (median and interquartile range, IQR) in the co‐culture media of DDFT‐NBF experimental groups media alone (media), media containing 2 × 10^9^ ± 0.1 × 10^9^ particles/mL or 10 μg/mL BM‐EV (BM‐EV), 10 ng/mL IL‐1β (IL‐1β) or IL‐1β + BM‐EV. Significant differences between experimental groups depicted via bar and asterisk.

The median and IQR of TNF‐α and IL‐6 co‐culture media concentrations of the experimental groups are depicted in Figure 3A,B, respectively. There was a trend to significant difference (*p* = 0.07) in the TNFα concentrations between the experimental groups. IL‐6 concentration in the media of BM‐EV (*p* = 0.02) and EV + IL‐1β (*p* = 0.03) treated DDFT‐NBF co‐cultures were significantly increased compared with the media control. There were no other significant differences between treatment groups.

### 3.5 ECM protective effect of BM‐EV on DDFT‐NBF co‐cultures

**FIGURE 4:**
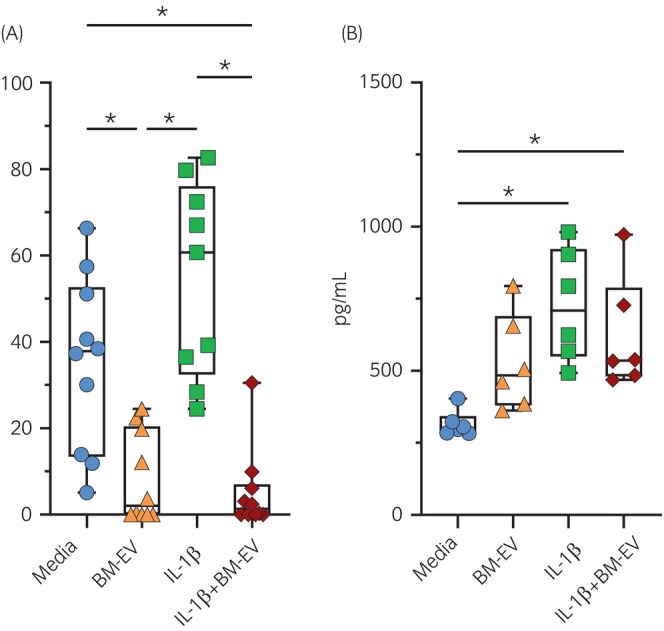
(A) MMP‐3 and (B) MMP‐13 concentrations (median and interquartile range, IQR) in co‐culture media of DDFT‐NBF experimental groups media alone (media), media containing 2 × 10^9^ ± 0.1 × 10^9^ particles/mL or 10 μg/mL BM‐EV (BM‐EV), 10 ng/mL IL‐1β (IL‐1β) or IL‐1β + BM‐EV. Significant differences between experimental groups depicted via bar and asterisk.

ECM degradation enzymes, MMP‐3 and MMP‐13 concentrations in co‐culture media are depicted in Figure 4A,B, respectively. MMP‐3 concentrations in BM‐EV‐ (*p* = 0.03) and BM‐EV + IL‐1β‐treated (*p* = 0.01) experimental groups were significantly lower than the media control. Additionally, the MMP‐3 concentrations in BM‐EV‐ (*p* = 0.006) and BM‐EV + IL‐1β‐treated (*p* = 0.002) groups were significantly lower than the IL‐1β‐treated group.

MMP‐13 concentrations in IL‐1β‐ (*p* = 0.002) and BM‐EV + IL‐1β‐treated (*p* = 0.02) groups were significantly increased compared with media control. There were no other significant differences between remaining experimental groups.

**FIGURE 5:**
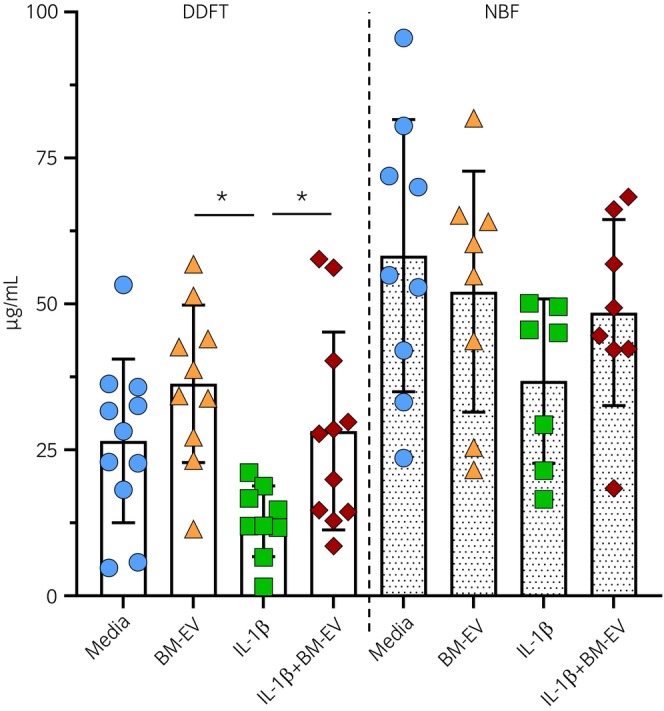
sGAG content in co‐cultured DDFT and NBF explants maintained in media alone (media), media containing 2 × 10^9^ ± 0.1 × 10^9^ particles/mL or 10 μg/mL BM‐EV (BM‐EV), 10 ng/mL IL‐1β (IL‐1β) or IL‐1β + BM‐EV. Significant differences between experimental groups depicted via bar and asterisk.

The sGAG content of DDFT and NBF explants was normalised to the explant DNA content and measured with DMMB assay. The data were normally distributed. The sGAG content of the NBF explants did not vary between treatments (*p* = 0.4) (Figure 5). The sGAG contents of BM‐EV‐ (*p* = 0.003) and BM‐EV + IL‐1β‐treated (*p* = 0.002) DDFT explants was significantly higher than the IL‐1β‐treated DDFT explants (Figure 5B). There were no other significant differences between remaining experimental groups.

## 4 DISCUSSION

Mesenchymal stem cell‐derived EV are increasingly evaluated as a novel therapy for a wide range of musculoskeletal conditions. Optimising equine BM‐EV preparation and evaluating the constituent bioactive molecules are essential prerequisites for downstream applications. In this study, equine BM‐EV of similar size and concentration were successfully isolated from BM‐MSC culture media via ultracentrifugation and precipitation methods. IL‐6, TGFβ1, PDGF and VEGF were quantified in equine BM‐EV. Although large intersample variabilities in IL‐6, TGFβ1, PDGF and VEGF concentrations were noted in equine BM‐EV isolated via ultracentrifugation, there were no significant differences in the cytokine and growth factor concentrations of equine BM‐EV isolated via ultracentrifugation and precipitation methods. Ultracentrifugation is the gold standard methodology for EV isolation; EV precipitation via a commercially available reagent was also selected as this protocol is feasible with a high‐speed centrifuge. To our knowledge, this is the first report on equine BM‐EV cytokine and tissue anabolic growth factor profiles.

Consistent with the results of this study, multi‐factorial cytokine IL‐6, which exhibits pro‐ and anti‐inflammatory, immunomodulatory, cell migratory and proliferative effects, was also enriched in rat and human BM‐EV.^46^, ^47^ Both IL‐6 and TGFβ1 gene expression and secretion were documented in rat BM‐EV and promoted neuronal regrowth after spinal cord injury.^48^ The targeted cytokine and growth factor quantifications conducted in this study demonstrates pro‐angiogenic and immunomodulatory benefits of equine BM‐EV, and follow‐up miRNA and proteomic analyses can be useful to further define equine BM‐EV small molecule protein cargo that can be harnessed for musculoskeletal regeneration.

To assess the anti‐inflammatory and ECM protective potentials of equine BM‐EV in the context of navicular disease, an in vitro explant co‐culture model that facilitates crosstalk between DDFT and NBF tissues was used.^49^ IL‐1β successfully induced an in vitro pro‐inflammatory environment causing ECM breakdown as demonstrated by co‐culture media MMP‐3 and MMP‐13 increase and explant sGAG content decrease relative to the media control group. Although the media concentration of TNFα in IL‐1β‐treated co‐culture was higher, it did not significantly differ between the experimental groups of this study. In the presence of IL‐1β inflammatory stimulus, BM‐EV treatment exhibited anti‐inflammatory and ECM protective effects that were evident as increased IL‐6, decreased MMP‐3 concentrations in the co‐culture media, as well as increased sGAG content of DDFT explants. Corroborating the results of this study, increased IL‐6 gene expression in inflamed equine and human chondrocytes following in vitro BM‐EV treatment has been previously reported.^30^, ^50^ The significantly increased co‐culture media concentration of IL‐6 in BM‐EV + IL‐1β‐treated DDFT‐NBF explants is likely in part a reflection of IL‐6 enrichment in BM‐EV isolates. Accepting the multifactorial role of IL‐6 and prior in vitro studies where in IL‐6 exposure of chondrocytes and synoviocytes has induced MMP and tissue inhibitors of MMP (TIMP),^51^, ^52^ quantifying media TIMP‐1 and ‐2 concentrations may be beneficial for clarifying the anti‐inflammatory status of BM‐EV treated DDFT‐NBF co‐cultures.

Decrease in co‐culture media MMP‐3 levels in BM‐EV‐ and BM‐EV + IL‐1β‐treated groups was the most promising indicator of BM‐EV ECM protective effect on DDFT and NBF tissues. Although the median MMP‐13 concentration in BM‐EV + IL‐1β‐treated groups was ~50% lower than the IL‐1β‐treated group, this difference was not statistically significant, and is likely due to the large intersample variability. In a recent in vitro study evaluating the anti‐inflammatory effects of equine BM‐EV on metacarpo‐/tarsophalangeal chondrocytes exposed to IL‐1β, MMP‐13 gene expression was also significantly decreased with BM‐EV treatment.^30^ Furthermore, BM‐EV treatment has exhibited anti‐collagenase activity in the contexts of human osteoarthritic chondrocytes in vitro inflamed with TNF‐α and during in vivo collagenase‐induced murine osteoarthritis.^50^, ^53^ Collectively, existing evidence and the results of this study support the anti‐inflammatory and ECM protective effects of murine, equine and human BM‐EV, and also lends credence to an EV‐mediated mechanism of action for decreased MMP when in vitro inflamed chondrocytes are treated with BM‐MSC conditioned media.^29^

To evaluate the effect of equine BM‐EV on navicular tissue ECM, the sGAG content of DDFT and NBF explants was assessed. The sGAG contents of BM‐EV‐ and BM‐EV + IL‐1β‐treated DDFT explants were significantly higher compared with IL‐1β‐treated DDFT explants. EV can promote ECM synthesis via cell proliferation and migration.^29^ Human BM‐EV isolated via UCF induced aggrecan gene expression and increased proteoglycan and collagen type II secretions from OA chondrocytes.^50^ Concurrent assessments of ECM synthesis in DDFT and NBF explants during co‐culture and in tandem with tissue inhibitors of MMP (TIMP) secretion in the co‐culture media will help further clarify ECM secretion and remodelling effects of BM‐EV.

There are limitations of this in vitro study to consider. This in vitro navicular tissue co‐culture model does not replicate in vivo conditions seen in clinical cases. BM‐EV and IL‐1β were introduced simultaneously to the experimental groups, whereas, in clinical cases, the inflammatory stimulus precedes therapeutic intervention. Although IL‐1β concentration used in this study has been reported previously with cartilage and chondrocyte studies^54^, ^55^; the role of IL‐1β alone as the inflammatory mediator in tendonitis/bursitis is not well‐defined and may be excessive relative to what is encountered clinically. A single 10 μg/mL dose of BM‐EV used concurrently with IL‐1β treatment was evaluated for its anti‐inflammatory and ECM protective properties. Future studies that explore the dose‐dependent effects of BM‐EV, along with sequential BM‐EV treatment following an inflammatory stimulus are warranted prior to in vivo equine BM‐EV assessments. Finally, including non‐MSC‐derived EV as an EV control treatment group may help clarify if the observed effects were due to BM‐EV or would be seen with EV in general. From a navicular tissue specific standpoint, the co‐culture analyses were limited by the number of tissues/explants that can be harvested from the forelimbs of a horse. Given the considerable variability between horses and a larger sample size may be warranted for comprehensive assessment of BM‐EV therapeutic efficacy.

For treating experimental and clinical tendon and cartilage pathologies in horses, BM‐MSC possess anti‐inflammatory properties, stimulate anabolic effects on matrix synthesis, improve return to athleticism and tissue histological structure.^14^, ^15^, ^16^, ^56^ In vivo cell‐tracking studies have demonstrated that very few MSC remain at the location of injection in horses (both tendon and joint).^18^, ^57^ This indicates that their therapeutic impact likely occurs during their transient presence at the site of injury. Local cytokine modulation and trophic factor synthesis have been proposed as possible mechanisms by which MSC influence tissue regeneration.^20^, ^58^, ^59^ Therefore, harnessing the beneficial properties of MSC, while escaping the constraints of extended in vitro culture time, donor specificity or autogenous cells and immunogenicity of allogeneic cells would be of great clinical utility. Evidence is mounting that EV are the mechanism by which the positive effects of MSC can be obtained while minimising delay in treatment and risk to the patient. As stable storehouses of growth factors and cytokines that are free of cellular material, EV mitigates immunogenicity concerns and promotes healing. Moreover, EV could be generated and stored for clinical use from pooled samples of donor MSC. Additional work investigating the effects of EV isolated from a different cell type and source on navicular tissues is warranted.

In conclusion, this research lays a foundation for future work to evaluate BM‐EV as an ‘off‐the‐shelf’ intra‐bursal/intra‐synovial therapy for horses diagnosed with navicular pathologies. Anti‐inflammatory effects of BM‐EV were most pronounced in the reduced MMP‐3 levels, while ECM preservation was demonstrated in the DDFT tissue. Further investigations are needed to optimise the BM‐EV dose and to assess their function in in vivo experiments prior to testing in naturally occurring disease. The data presented here support the need for such further endeavours.

## FUNDING INFORMATION

ACVS Foundation 2019 Surgeon‐in‐Training Grant Program and, in part, by the National Center for Advancing Translational Sciences of the National Institutes of Health under Grant Number KL2TR002734.

## CONFLICT OF INTEREST STATEMENT

No competing interests have been declared.

## AUTHOR CONTRIBUTIONS

**Vivian Quam:** Data curation; formal analysis; funding acquisition; investigation; writing – original draft; writing – review and editing. **Zarah A. Belacic:** Data curation; formal analysis; investigation; methodology; writing – review and editing. **Sidney Long:** Data curation; formal analysis; investigation. **Hilary C. Rice:** Data curation; writing – review and editing. **Madhu S. Dhar:** Conceptualization; investigation; methodology; writing – review and editing. **Sushmitha Durgam:** Conceptualization; data curation; formal analysis; funding acquisition; investigation; methodology; project administration; supervision; validation; writing – original draft; writing – review and editing.

## DATA INTEGRITY STATEMENT

Sushmitha Durgam confirms full access to all the data in the study and takes responsibility for the integrity of the data and the accuracy of the data analysis.

## ETHICAL ANIMAL RESEARCH

The experimental protocol was approved by the Institutional Animal Care and Use Committee.

## INFORMED CONSENT

Tissues were collected from horses donated for unrestricted research use.

### PEER REVIEW

The peer review history for this article is available at https://www.webofscience.com/api/gateway/wos/peer-review/10.1111/evj.14090.

## Supporting information


**Table S1.** Signalment of horses from which sternal bone marrow aspirates were collected for BM‐MSC was isolated for BM‐EV preparation.

**Table S2.** Signalment of horses from which DDFT and NBF explants was obtained.

## Data Availability

The data that support the findings will be available in FigShare at [doi:10.6084/m9.figshare.25505161] following an embargo from the date of publication to allow for commercialisation of research findings.
