# Supplementary material for: Equine bone marrow MSC‐derived extracellular vesicles mitigate the inflammatory effects of interleukin‐1β on navicular tissues in vitro
Source: Equine Vet J. 2024 Apr 8;57(1):232–42. doi: 10.1111/evj.14090 (PMC11458820; doi:10.1111/evj.14090)
Supplement: Supplementary file 1 — Table S1. Signalment of horses from which sternal bone marrow aspirates were collected for BM‐MSC was isolated for BM‐EV preparation. [file EVJ-57-232-s002.pdf]

Equine Veterinary Journal BEVA

Table S1: Signalment of horses from which sternal bone marrow aspirates were collected for BM-MSC were isolated for BM-EV preparation.

Horse	Age (years)	Sex	Breed	Health Status
1	4	Gelding	Mixed breed	Healthy
2	4	Gelding	Quarter Horse	Euthanised for distal limb laceration involving tendon sheath
3	5	Gelding	Quarter Horse	Healthy
4	8	Female	Mixed Breed	Healthy
5	8	Gelding	Quarter Horse	Healthy
6	12	Female	Appendix	Euthanised for strangulating intestine-related colic
7	12	Gelding	Quarter Horse	Healthy

Five horses (#1,3,4,5,7) were from the University teaching and research herd, and the others were sampled (prior to euthanasia) following informed consent from clients.
