# Supplementary material for: Equine bone marrow MSC‐derived extracellular vesicles mitigate the inflammatory effects of interleukin‐1β on navicular tissues in vitro
Source: Equine Vet J. 2024 Apr 8;57(1):232–42. doi: 10.1111/evj.14090 (PMC11458820; doi:10.1111/evj.14090)
Supplement: Supplementary file 2 — Table S2. Signalment of horses from which DDFT and NBF explants was obtained. [file EVJ-57-232-s001.pdf]

Equine Veterinary Journal BEVA

**Table S2:** Signalment of horses from which DDFT and NBF explants were obtained.

| Horse | Age (years) | Sex | Breed | Health Status |
|---|---|---|---|---|
| 1 | 4 | Gelding | Thoroughbred | Euthanised due to cervical compression |
| 2 | 4 | Gelding | Quarter Horse | Euthanised for hind distal limb laceration involving tendon sheath and flexor tendons |
| 3 | 5 | Gelding | Quarter Horse | Healthy |
| 4 | 8 | Female | Mixed Breed | Healthy |
| 5 | 8 | Gelding | Quarter Horse | Healthy |
| 6 | 12 | Female | Appendix | Euthanised for strangulating intestine-related colic |
| 7 | 12 | Gelding | Quarter Horse | Healthy |

Four horses (#3,4,5,7) were from the University teaching and research herd that were euthanised for the purpose of reducing herd size due to budget restrictions related to the pandemic. Sternal bone marrow aspirates were sampled prior to euthanasia as stated previously from the same horses. Others were sampled (prior to euthanasia) following informed consent from clients.
